# Barrier analysis for continuity of palliative care from health facility to household among adult cancer patients in Addis Ababa, Ethiopia

**DOI:** 10.1186/s12904-023-01181-w

**Published:** 2023-05-12

**Authors:** Yonas Abate, Kalkidan Solomon, Yoseph Mamo Azmera, Marlieke de Fouw, Mirgissa Kaba

**Affiliations:** 1 School of Public Health, Department of Preventive Medicine, Addis Ababa University, Addis Ababa, Ethiopia; 2 Hospice Ethiopia, Addis Ababa, Ethiopia; 3 Department of Gynecology, Leiden University Medical Center, Leiden, The Netherlands

**Keywords:** Palliative care, Continuum of care, Barriers to palliative care, Cancer, Ethiopia

## Abstract

**Background:**

Annually 57 million people across the globe require palliative care, 76% are from low- and-middle income countries. Continuity of palliative care contributes to a decline in emergency room visits., decreased hospital deaths, improved patient satisfaction, better utilization of services, and cost savings. Despite efforts made to develop the palliative care guideline in Ethiopia, the service is not yet organized and linked to primary health care. This study aimed to explore barriers to the continuum of palliative care from facility to household for cancer patients in Addis Ababa.

**Methods:**

Qualitative exploratory study was conducted with face-to-face interviews with a total of 25 participants. The study population was adult cancer patients, primary caregivers, healthcare providers, volunteers, and nationwide advocates. Data were audio recorded, transcribed verbatim and finally imported to Open code version 4.02 software for coding and analysis. Thematic analysis was guided by Tanahashi’s framework.

**Results:**

The key barriers to continuity of palliative care included opioid scarcity and turnover and shortage of healthcare workers. A shortfall of diagnostic materials, cost of medications, lack of government backing, and home-based center’s enrollment capacity hampered accessibility. Care providers were instruments of cultural barriers in delivering appropriate end-of-life care, on the other hand, patients’ preference for conventional medicine hindered acceptability. Lack of community volunteers, failure of health extension workers to link patients, and spatial limits fraught utilization. The lack of defined roles and services at several levels and the workload on healthcare professionals affected the effectiveness of the nexus.

**Conclusion:**

The continuum of palliative care service from health facility to household in Ethiopia is yet in its infancy compromised by factors related to availability, accessibility, acceptability, utilization, and effectiveness. Further research is required to delineate the roles of various actors; the health sector should smudge out the continuum of palliation to cope with the growing need for palliative care.

**Supplementary Information:**

The online version contains supplementary material available at 10.1186/s12904-023-01181-w.

## Background

Cancer is an important clinical and public health problem worldwide. In 2020, 19.3 million new cancer cases and 10.0 million cancer deaths were reported by the Global Organization Board Of Cancer Association Network (GLOBOCAN) [2]. Approximately 70% of deaths from cancer occur in low and middle-income countries (LMICs) [3].

The World Health Organization (WHO) defines Palliative Care (PC) as an approach that improves the quality of life of patients and their families facing problems associated with life-threatening illnesses, through the prevention and relief of suffering through early identification and impeccable assessment and treatment of pain and other problems, both physical, psychosocial and spiritual [4].

Every year approximately 56.8 million individuals worldwide require palliative care, with 76% coming from low- and middle-income countries and 67.1% being adults. Only around 14% of patients who require palliative care receive it [5]. Over 80% of advanced cancer patients will benefit from relatively simple and low-cost palliative care interventions that can be integrated into primary health care and home care services [6].

Three interrelated dimensions of continuity of care have been stated. PC should be provided through an integrated, collaborative and community resource-linked continuum of care, which focuses on Home-Based Palliative Care (HBPC). HBPC is one of the models that help to increase accessibility, and continuity and ensure sustainability [7].

Continuity of care is associated with lower rates of emergency department visits, decreased hospital deaths, improved patient satisfaction, better utilization of services, and significant cost savings with palliative care needs. being met [8, 9].

Lack of continuity of PC can cause undesired experiences such as feeling unsafe during illness transitions, patients being transferred between multidisciplinary teams, and suboptimal support for problems and needs [10]. PC cannot be developed without achieving integration for sustainable development. One in every three major barriers to PC development is related to the lack of integration within the health system. The barriers are a lack of health policies in support of palliative care development, a lack of relevant training for healthcare workers, and poor accessibility of essential palliative care drugs [4].

With the immense need for PC and at the same time insufficient palliative care services, limited availability of drugs and other resources, and lack of trained palliative care professionals combined with the high burden of non-communicable diseases, the demand for palliative care services far exceeds the supply [11].

The government of Ethiopia has developed a national PC guideline, however, the service is not fully integrated at the primary healthcare level and there are barriers to the continuity of palliative care for cancer patients. To identify bottlenecks in the operation of the service, to analyze the constraining factors responsible for such bottlenecks, and to select effective measures for service development studies that aim to assess barriers to effective coverage with health services uses the Tanahashi framework. The framework explores the barriers to coverage of health services in five different dimensions. i.e. availability, accessibility, affordability, contact, and effectiveness [12].

Therefore, this study aimed to explore barriers to the continuum of palliative care using the Tanahashi framework from facility to household among cancer patients receiving palliative care in Addis Ababa, Ethiopia.

## Methods

### Study area and period

The study was conducted from 01 to 2020 to 30 September 2020 in Addis Ababa, the capital city of Ethiopia in two hospitals, Tikur Anbessa and Yekatit hospital, and, Hospice Ethiopia. Tikur Anbessa (TASH) is the tertiary referral hospital in Ethiopia, where cancer treatment and PC services practically exist. This hospital has 5 beds devoted to palliative care and administered by Addis Ababa University. Yekatit 12 memorial hospital is one of the largest specialized hospitals in Addis Ababa and has provided palliative care for seriously ill patients for the past three consecutive years. During the same period, Yekatit hospital provided palliative care for seriously ill patients, including cancer patients.

Hospice Ethiopia is a non-governmental organization based in Addis Ababa. Hospice Ethiopia provides care to people with serious illness and their families, raises awareness of healthcare workers in palliative care, advocates for palliative care, and creates an enabling environment for providing palliative care in Ethiopia.

In TASH, Yekatit 12 memorial hospital, Hospice, and the Federal Ministry of Health (FMoH) 1–2 trained staff are working on palliative care in each institution. In the hospitals, the same staff who works on cancer care works on palliative care.

### Study approach

An exploratory qualitative study with a deductive approach was used while interviewing patients, caregivers, and professionals and their views on the patient-professional interaction and the organization of the healthcare system [13, 14].

### Study population

Study participants were patients with their primary caregivers, volunteers, health care providers (nurses, physicians, and specialists), and nationwide palliative care advisors/advocators. We included patients who were 18 years old and above, diagnosed with cancer, and informed about their illness, those who were able to give consent, and patients who received palliative care service. Caregivers who were daily involved in providing care to the patient were also enrolled. The patients and caregivers were selected by the health professionals in the facilities and the health care providers and national advisors were selected purposely by the PI based on their involvement and background related to our topic of interest. Based on Creswell, J. W recommendation for qualitative studies we initially planned to enroll 20–30 participants [15]. However, saturation was achieved after 22 participants were interviewed. Additional 3 participants from each group were interviewed after presumed saturation was achieved to ascertain the repetition of evidence.

### Selection of study participants

Participants were purposefully enrolled. Maximum variation and gradual selection were applied. Maximum variation assisted us to look at different perspectives from diverse participants and gradual selection aided the study to incorporate information-rich participants. We included patients from both rural and urban areas, with different cancer diagnoses, and key informants from both government hospitals and NGOs with different levels of experience in palliative care. The interviews were directed through probing questions.

### Data collection tool and procedures

Participants were interviewed in the Amharic language, in a location of their choice. After reviewing the literature our prompt and other parts of open-ended interview guides were developed based on the umbrella of the Tanahashi framework.

Interview guides were developed to gather detailed information on socio-demographic characteristics, and questions about diagnosis, barriers to palliative care, continuum of care, and areas of improvement [see Additional file 1–4] It was prepared in English and translated into the Amharic language by a professional translator. The translation was cross-checked by two healthcare professionals independently. Two male data collectors with a first degree in public health and who had experience in qualitative research conducted the interviews.

Nine cancer patients and seven primary caregivers were involved in the in-depth interviews to generate evidence of their individual experiences. Additionally, we conducted two Key informant interviews from TASH, one from Yekatit 12 hospital, four from Hospice Ethiopia, and two with national-level advisors and advocators at the Ministry of Health and they responded to what is believed to shared information on barriers to the continuum of palliative care from facility to household for cancer patients. We only conducted individual interviews because of the situation of COVID-19.

The interviews were pre-arranged, led by the researchers and all interviews were conducted face to face at the venue of choice of participants. Memos and scribbles were noted during the interview by the interviewers. The interview guide was continuously modified throughout the data collection to include newly occurring issues and to improve the clarity of the interview questions. The duration of each interview ranged from 30 to 50 min.

### Data management and analysis

Tanahashi’s framework was applied to illustrate the coverage of PC in this study; it has stages that have five important steps. These are availability, accessibility, affordability, contact, and effectiveness [12].

After finishing each interview session, information stored on an audio recorder was preliminarily analyzed, and the participant’s description of issues was internalized and transcribed verbatim until saturation was reached. The audio-recorded interviews were transcribed in Amharic, translated into English, and aligned with the field notes and memos. A codebook was developed after interviewing a few study participants to guide the researcher’s coding consistency throughout the data analysis process. Emerging ideas during the interview were added to the codebook and similar ideas were combined with earlier codes.

Researchers coded data and in case of discrepancies, KS and MK were involved to verify and reach a decision. Data analysis was facilitated by Open-code version 4.02. Before the analysis, a consensus was reached among the team on the coded themes and subthemes.

Deductive approaches were followed to analyze and interpret the findings in line with the Tanahashi framework to classify, organize and attach meaning to the data according to the key themes, concepts, and emerging categories (Table 1). The results were presented as themes with supportive verbatim quotes.

**Table 1:** List of themes, categories, and codes

Themes	Categories/codes
Availability	• Resource o Turnover of staff o Trained personnel o Workload o Medications (pain relief) o Equipment
Accessibility	• Physical access o Distance from and to a health facility o Cost of service o Infrastructure o Waiting time
Acceptability	• Willingness to use o Conventional medicine o BUY-in from Hospitals o Culture
Contact	• Continuity of care o Policy o Insufficient discussion time with healthcare professionals o Lack of connectors o Service confined to limited facility
Effectiveness	• Quality and effectiveness of service o Focusing on Physical symptom o Focusing on treatment o Not involving family

### Data quality assurance

The investigators first got familiarized with the study setting and established a close and friendly relationship by conducting a repeat visit to entree real information and constancy. In addition to the initial training, the data collectors were closely supervised by the principal investigator. Interviews were conducted in settings preferred by the patient, caregiver, or healthcare provider to ensure a comfortable environment for discussion. Notes taken during the interview and the verbatim transcription were saved for verifying the process and to endure the consistency of the interpretations.

The similarity of the codes was checked by the code-recode strategy. The researchers guaranteed confirmability through the words of study participants, peer review, and data were triangulated by method, place, and individual participant’s profile. An effort was made to maintain the original meanings when interpreting the data.,

## Results

### Socio-demographic characteristics of participants

Table 2 illustrates the characteristics of the study participants. We enrolled 25 participants in total, of which 16 participants for in-depth interviews including 9 patients and 7 primary caregivers. Their age was in median age was 40 IQR ± 15.75 years,10 were female, four were unemployed and 2 had no formal education we conducted 9 key informant interviews with healthcare providers, advocators, and volunteers. The age of the key informants ranged from 30 IQR ± 17.5 years, and 4 were government employees (Table 2). The health care providers provide PC either home to home or at the health facility, the two advocates work closely with FMOH as consultants and advocates of PC. The volunteers are people from the community that help the hospice center get in touch with a patient in need from the community.

**Table 2:** Socio-demographic status of participants

		IDI (n = 16)	KII (n = 9)
Age			
		Median IQR 40 IQR ± 15.75	Median IQR 30 ± 17.5
		**Frequency**	**Frequency**
Sex			
	Male	6	3
	Female	10	6
Marital status			
	Never married	5	4
	Married	9	5
	Widow	2	-
Educational status			
	No formal education	2	-
	Can read and write	1	-
	Primary	2	-
	Secondary	5	-
	Diploma and above	6	9
Employment status			
	Government employee	4	4
	Private work	3	3
	Housewife	5	-
	Unemployed	4	-
	Volunteers	-	2
Type of cancer			
	Anal cancer	1	-
	Cervical cancer	2	-
	Gastric cancer	1	-
	Throat cancer	2	-
	Sarcoma	1	-
	Breast cancer	1	-
	Unspecified cancer	1	-

### Thematic analysis

#### Availability

Key informants identified turnover of health care staff, turnover of chief executive officers (CEOs), and lack of attention from higher officials as the main barriers to the continuum of palliative care.

“… When working with government hospitals continuity is always a challenge; the turnover of personnel is mind-boggling; we try to train and establish a solid palliative care team, and then two years later it’s different staffs and different CEOs; however, as more people are taught, the more consistency comes.“ (52 years old female advocator)

In addition, all key informants observed a lack of educated and trained personnel as a barrier to the continuity of care; one of the advocators stated

“It is necessary to build capacity and teach palliative care to doctors and nurses in undergraduate and postgraduate programs to maximize integration into their daily practice and community involvement.“ (55 years old, male advocator)

Most study participants agreed that late diagnosis and the absence of diagnosing instruments were major challenges for them. As patients were not diagnosed earlier, they won’t get palliative care service as soon as they deserve. One female caregiver said…

“She went first to a health center and other clinics but her illness was not diagnosed earlier the tumor stayed and reached an advanced stage. Therefore, the medical instrument that is needed for the diagnosis of cancer should be available; they were giving her simple medication saying that it is just her menstrual cycle for a long time.” (45 years old female caregiver).

All key informants and four patients stated that there is a major gap in the availability of medication. Morphine is the most important medication in short supply.

“We used to produce oral morphine suspensions by importing the powder, but scarcities appeared as supply fell again due to lack of ownership and the initiative’s donor-driven structure. Immediately we began importing from Switzerland to provide those hospitals who were in desperate need of it.“ (55 years old male advocator)

“Even if I had money to buy the medications; stock out is the main problem I faced most of the time…. (49 years old male throat cancer patient)

#### Accessibility

Rural participants stated that palliative care is only available at the healthcare facility and is limited to a specific day of each week, i.e. Tuesday and Thursday, and that the service is not accessible in their community. Furthermore, most patients cited transportation costs to the healthcare facility as a barrier to receiving palliative care.

“On one occasion, I arrived late at the hospital on Tuesday and was told to return on Thursday because the doctor had already left; as a result, I had to spend extra days at extra expense until Thursday to avoid traveling 228 kilometers back home.“ (30-year-old male cancer patient)

Patients and key informants brought up infrastructure issues, such as the limited human resource capacity to enroll all patients in the home-based service, which limits and delays the continuum of care.

“...let alone offering us the service at home, it was enough if we got the service at the zonal hospital; we’re dying waiting for our appointment ...“ (45-year-old male cancer patient)

Patients, caregivers, and health care providers believed that late diagnosis and lack of diagnostic devices were significant obstacles for them. As a result, palliative care is not integrated earlier.

“She first went to a health center, but the tumor remained and progressed to advanced stage because her sickness was not recognized sooner; they had been providing her modest medication for a long time, claiming it was a menstrual cycle.“ (45 years old female caregiver)

Caregivers responded that because of long waiting times for diagnosis and treatment cancer patients are dying without getting palliative care or any care at all. One caregiver said as below.

I think it is good if hospitals like TASH and Minilk give this service, there is a shortage of health care providers like doctors it is hard to have 2–3 doctors for all these patients I think that is why there is a long waiting time. There are people I know that died while waiting to be called at the hospital (28 years old female caregiver)

#### Acceptability

Culture plays a critical role in how patients, families, and healthcare providers view the end of life. Cultural competence in end-of-life care includes knowledge and experience and working in cross-cultural situations. Ethiopian culture may also be a barrier to palliative care. Healthcare providers expressed their concern about providing palliative care, particularly end-of-life care, and dealing with dying cancer patients at home. The healthcare providers said that it is not acceptable to talk about death in the Ethiopian culture. A female health worker said the following:

“...it is quite stressful; always dealing with a terminally ill person is tough for us culturally to cope with such a circumstance and discuss the end of life care” (30 years old female care provider)

Advocators mentioned patients’ preference for conventional medicine as a barrier to palliative care services, and patients end up withdrawing from home-based palliative care programs.

“One of the issues with cancer patients is that when they discover a lump or whatever, they go to a traditional healer or a priest or go to Tsebel (holy water), and then it takes them longer to go to a medical center and see an oncologist in time.“ (52 years old female advocator)

On the other hand, a patient who lost his one leg due to a wrong procedure (in the hospital?) mentioned utilization of conventional medicine after giving up on the health system.

“Since I gave up on the health system, I’ve just used prayer and traditional treatment for the last six months; as a result, my condition deteriorated, and I was in excruciating pain ... (39 years old male unspecified cancer patient)

Even though hospitals and care providers desire to provide palliative care to patients, they need support from the government and other stakeholders to get recognized such as getting palliative care training to have a dedicated staff and avoid turnover, availability of diagnostic and treatment for cancer in-addition availability of strong anti-pains. Government and hospitals have not recognized and buy-into availing of the palliative care service. One healthcare provider stated

“Unless policymakers and higher authorities take a reasonable approach to this, they will not allow us to work; they will put a stop to it; as a result, palliative care has received insufficient government attention, which is a huge hurdle.“ (A 33-year-old male healthcare professional).

#### Contact

Patients complained about not having enough time to talk with their doctors. If patients and caregivers from their families are not given adequate time and space for discussion, this could be a massive obstacle to palliative treatment.

“Certainly, there isn’t enough time; they just call you by name, you step in, they write you a prescription and ask you a few things, and then the next person is called. There will be no discussion.“ (30 years old male cancer patient).

Besides, palliative care is confined to specific days in TASH, as mentioned in the section about the availability of services. Even patients misinterpreted the notion of the service with the names of specific days, so most patients know palliative care as ‘the Tuesday and Thursday service.’ A cervical cancer patient stated her feelings as follows:

“The service that I received on Tuesday will not be available on other working days. I’m sure they’ll instruct me to return on those particular days two weekdays if I have any other symptoms.“ (42-year-old female cervical cancer patient)

Furthermore, healthcare providers stated that the service should be expanded to meet the needs of the entire population, but that there is a shortage of connectors, that health extension workers are overwhelmed with many activities and become ineffective, lack of government and public commitment in addition volunteerism is not a culture.

“...human resources are required to enable the link. A volunteer from one cluster is not supposed to work in another, so we need to recruit and train different volunteers from different clusters to improve the linkage.“ (A 32-year-old female health care provider)

There is no established structure for implementing palliative care continuity from facility to home, and policy instructions and guidance are lacking. There is a lack of awareness and carelessness of healthcare providers is the reason I think the chain does not exist. If hospice and hospitals have a link and if there is a way, we exchange patients that would be good. (25-year-old health care provider)

#### Effectiveness

All healthcare providers from the hospitals were raising the issue of a high workload which leads to only treating the physical symptoms of patients by ignoring the other components of palliative care and not linking the patients to home-based care centers.

“...speaking the truth, it is difficult for us to manage given the number of patients and the pressure of work, so we are focusing on managing physical symptoms rather than devoting time to psychosocial and spiritual components.“ (30 years old health care provider)

Furthermore, healthcare providers explained that palliative care is currently provided in a generalized manner in which one provider supports a patient’s physical, psychological, social, and spiritual needs all at once, which leads to ineffective service delivery and forces healthcare providers to focus solely on the physical component. According to a 29-year-old healthcare worker mentioned,

“Due to several factors such as drug availability, human resource, and financial constraints, we are now simply supporting patients’ physical needs; nonetheless, it would have been preferable if the complete package of palliative care services were offered.” (30-year-old, female, health care provider)

Focusing on treating or prescribing drugs and considering palliative care as a luxury were among health care providers’ major concerns raised from the side of health care providers as a barrier to palliative care. One healthcare provider said;

Even at the institution’s level, they only focus on the disease, not on the status of the patient’s pain and suffering. They consider proving palliative care as additional luxury care. In general palliative care in a country, the level is not much-practiced too much work is needed for the health workers.” (28 years old female health care provider).

Patients stated how important it is to involve family members in discussions about palliative care visits because it is a key aspect that should not be overlooked. A cervical cancer patient stated;

“.... my husband doesn’t attend my follow-up; I wish he did so that he could understand and be aware of my predicament, but he is naïve and still expect me to be sexually active, Unless the council session invites/oblige our partner is useless and incomprehensible.“ (42 years old female cervical cancer patient)

## Discussion

This study explored barriers to the continuum of palliative care from facility to household for cancer patients in Addis Ababa. Shortage of opioids, shortage of palliative care professionals, lack of attention from higher officials, lack of access, and defined structure for continuity of palliative care core barriers. In addition, physical accessibility of palliative care, transportation, and waiting time was detailed as a barrier. The lack of enough volunteers and overwhelmed health extension workers in the community annihilates the nexus of the service.

There should be a definite state of roles of various stakeholders at the community and facility levels to create a continuum of care for palliative care services to address the growing issues of choric illnesses.

According to study participants, primary care is a key interface for palliative care because it is ultimately where patients present initially with complaints or concerns.

PC should be accessible to the majority of the target population and carried out equitably across all levels of care, whether public or private; over 80% of advanced cancer patients will benefit from relatively simple and low-cost interventions that can be integrated into primary healthcare and home care services [6].

The other barrier mentioned was the availability of morphine which is the drug of choice for pain management for cancer patients. In our study, lack of access to pain medication and other essential medicines was repeatedly raised as a barrier for the continuum of PC. This finding is consistent with studies done in Ethiopia and Africa which reported access to opioids is inadequate because of several factors such as legal and regulatory restrictions, cultural misperceptions about pain, inadequate training of healthcare providers, procurement challenges, weak health systems, and concerns about diversion, addiction, and misuse [16–19].

On the other hand, Uganda has pioneered the prescription of morphine by nurses [19]. This implies that concerns related to medication in the primary health care units can be halted by offering training for nurses on how and to whom to prescribe opioid analgesics and improving the legal and regulatory policy restrictions on opioids.

In Ethiopia, let alone the continuum of care, palliative care in itself is a relatively new notion. In this study, some providers responded that providing PC is luxury care.

In a study done in Germany, palliative care education was integrated into the undergraduate curriculum as part of mandatory training and to get the license to practice medicine by legislation [20]. This indicates health care providers no longer consider PC as luxury care if palliative care was combined in the curricula of the current medical education system. Even though Germany is a developed country but the study still reflects that PC is not a luxury.

In our findings, conventional medicine and traditional healers are often visited by patients for curative service resulting in late diagnosis, and late recruitment to palliative care service. This finding is supported by a multi-country study done in Africa where seeking curative services for cancer from traditional healers and herbalists is common [18]. In Ethiopia, people have an exhaustive relationship with their religious and cultural healers.

Lack of access to palliative care is a major problem worldwide. In this study absence of palliative care services in rural facilities, and the inconvenient location and timing of service in urban settings hinder the physical accessibility of palliative care. Transportation was mentioned as the main barrier to the accessibility of palliative care provision. Palliative care coverage is particularly limited for patients in inaccessible geographical areas. This concept is parallel with studies done in Canada that reported distance and location in part determine the utilization of services and influence health outcomes [21, 22]. This implies that PC is affected by geography location thus people who were located closer to sites of care delivery are more likely to utilize the services because of reduced mobility and obtain better health outcomes and whereas an expression of pain is associated with weakness and higher waiting time for radiotherapy and chemotherapy may also lead them to this decision [23].

In our study lack of consistent communication between care providers with patients, lack of discussion time with patients, and not involving family members during discussion time were mentioned as a barrier. Similar to our findings other studies reported provider-related barriers such as providers not talking about patients’ problems, needs, and preferences as the disease progresses, difficulties of patients in dealing with the diagnosis denial, or hesitant attitude towards the prognosis [17]. This might be due to a structural lack of availability of the appropriate confidential room, work overload, and knowledge gap.

We have addressed the possible barriers to the continuity of palliative care from the perspectives of cancer patients and their caregivers, healthcare professionals, and advocates. Our findings, however, should be interpreted in light of some limitations. Because of the current situation of COVID, we were not able to do focus group discussions, as a result, we might have missed the group dynamic idea about barriers for the continuum of PC and were not able to fully explore different professional alliances and discrepancies on barriers for palliative care and nexus of the service. Our study only included cancer patients even though the findings might imply for all patients who might need palliative care; some of the findings might not apply to non-cancer cases or we might have missed some perspectives from the non-cancer cases.

## Conclusion

Our study addressed the lack of access to basic and specialized palliative care training and education for healthcare workers, limited opioid availability, costs for transportation and healthcare services, turn-over of trained staff, overwhelmed health workers, physical inaccessibility, and lack of a defined palliative care package as hindering factors for the continuum of palliative care service. We also discovered that there is no established structure for implementing palliative care continuity from facility to home, and that policy instructions and guidance are lacking.

We suggest that stakeholders such as pharmaceutical companies, and governmental and non-governmental organizations working on health work together to promote opioid analgesic availability and accessibility. Since there are bulky scarcities in skilled palliative care professionals within the study area, it is recommended to include palliative care in curricula and provide palliative care in collaboration with different hospitals on consistent and sustainable referral linkages to home-based palliative care services.

## Electronic supplementary material

Below is the link to the electronic supplementary material.

Additional file 1: Interview guide for patients. The interview guide includes questions on socio-demographic characteristics, and questions about diagnosis, barriers to palliative care, continuum of care, and areas of improvement.

Additional file 2: Interview guide for families/primary caregivers. The interview guide includes questions on socio-demographic characteristics, and questions about diagnosis, barriers to palliative care, continuum of care, and areas of improvement.

Additional file 3: Interview guide for healthcare providers. The interview guide includes questions on socio-demographic characteristics, and questions about diagnosis, barriers to palliative care, continuum of care, and areas of improvement.

Additional file 4: Interview guide for policymakers. The interview guide includes questions on socio-demographic characteristics, and questions about diagnosis, barriers to palliative care, continuum of care, and areas of improvement.

## Data Availability

The datasets used and/or analyzed during the current study are available from the corresponding author upon reasonable request.

## List of Abbreviation

FMoH: Federal Ministry of Health
EFCI: Female Cancer Initiative
GLOBOCAN: Global Organization Board of Cancer Association Network
HBPC: Home-Based Palliative Care
IDI: In-depth Interview
KII: Key Informant Interview
LMICs: Low and middle-income countries
NGO: Non-Governmental Organization
PC: Palliative Care
TASH: Tikur Anbessa Specialized Hospital
WHO: World Health Organization
