# Supplementary material for: Barrier analysis for continuity of palliative care from health facility to household among adult cancer patients in Addis Ababa, Ethiopia
Source: BMC Palliat Care. 2023 May 12;22:57. doi: 10.1186/s12904-023-01181-w (PMC10175902; doi:10.1186/s12904-023-01181-w)
Supplement: Supplementary file 1 — Additional file 1: Interview guide for patients. The interview guide includes questions on socio-demographic characteristics, and questions about diagnosis, barriers to palliative care, continuum of care, and areas of improvement. [file 12904_2023_1181_MOESM1_ESM.docx]

Interview guide for adult cancer Patients

1. Can you please introduce yourself? Probe: sex, marital status, education, employment, religion, and medical characteristics such as diagnosis, stage, and treatments you are taking?
2. Can you tell me about your diagnosis? Probe: When did the problems start? What happened next? Did you seek any advice? From whom?
3. Have you ever heard about palliative care? Probe: the term by itself, palliative care provision components, possible places of care?
4. Have you ever received home-based palliative care for yourself? Probe: if yes, what services did you receive, and who provides you service? When did you start taking the service?
5. What were the barriers for you to access palliative care services? Probe: from health facilities, from health care providers, from your perspective
6. What were the barriers while receiving home-based palliative care service? Probe: from health facilities, from health care providers, from your perspective?
7. Are you involved in the planning and decision process? Probe: Are you Informed and involved in care and treatment decisions? Is there appropriate space, accommodation, and time for interaction with healthcare providers? How? What do you think might have caused this?
8. Has your problem got worse/better after you started palliative care? How has this affected your day-to-day life? Probe: ask about activities of daily living (work; in the house; social activities) what do you like about palliative care service? Why?
9. Can you suggest how continuity of palliative care from health facility to household and from household to facility be insured?
10. Any other concerns you want to share with me or if there is anything you want to add?

**Thank you for your kind cooperation, I will re-visit you based on your willingness for missed or untouched issues if any.**
