# Supplementary material for: Barrier analysis for continuity of palliative care from health facility to household among adult cancer patients in Addis Ababa, Ethiopia
Source: BMC Palliat Care. 2023 May 12;22:57. doi: 10.1186/s12904-023-01181-w (PMC10175902; doi:10.1186/s12904-023-01181-w)
Supplement: Supplementary file 2 — Additional file 2: Interview guide for families/primary caregivers. The interview guide includes questions on socio-demographic characteristics, and questions about diagnosis, barriers to palliative care, continuum of care, and areas of improvement. [file 12904_2023_1181_MOESM2_ESM.docx]

Interview guide for Families/Primary caregivers

1. Can you please introduce yourself? Probe: sex, marital status, education, employment, religion, relationship with the patient
2. What do you understand when you hear the word PC?
3. Can you tell me how palliative care is appropriate for cancer patients? Probe: when do you consider the service should be provided? When did you decide to bring the patient to the hospital?
4. Are you able to respond to a patient’s palliative care needs? Probe: What, if any, challenges do you face when taking care of patients with cancer i.e. social, psychological, spiritual, and physical? Do you have a specific time or day to give this palliative care service?
5. Do you discuss with the patient you accompany and health care provider on preferred place of care? Probe: Are you Informed and involved in care and treatment decisions by the health care providers? Is there appropriate space, accommodation, and time for interaction with healthcare providers? What measures are taken to comply with these preferences?
6. Do you talk with patients about their spiritual or adequately attempt to assess patients’ access to spiritual and religious needs? Probe: how and when? What, if any, challenges do you meet?
7. What do you need to be able to increase your knowledge about Palliative care? How do you ensure proper support? What, if any, challenges do you meet?
8. What were the barriers for your ill family member to get palliative care services? Probe: from health facilities; from health care providers; from your perspective
9. What were the barriers for your ill family member to get home-based palliative care service? Probe: from health facilities; from health care providers; from your perspective; who facilitated for you to get this service;
10. Can you give me any suggestions on how to ensure the continuity of palliative care from the facility to Household and vise-verse?
11. Any other concerns you want to share with me or if there is anything you want to add?

**Thank you for your kind cooperation, I will re-visit you based on your willingness for missed or untouched issues if any.**
