# Supplementary material for: Barrier analysis for continuity of palliative care from health facility to household among adult cancer patients in Addis Ababa, Ethiopia
Source: BMC Palliat Care. 2023 May 12;22:57. doi: 10.1186/s12904-023-01181-w (PMC10175902; doi:10.1186/s12904-023-01181-w)
Supplement: Supplementary file 3 — Additional file 3: Interview guide for healthcare providers. The interview guide includes questions on socio-demographic characteristics, and questions about diagnosis, barriers to palliative care, continuum of care, and areas of improvement. [file 12904_2023_1181_MOESM3_ESM.docx]

Interview guide for healthcare providers

1. Can you introduce yourself for me? Probe: sex, marital status, educational status, employment, your work experience? Related with palliative care?
2. What do you understand when you hear the word Palliative care? Probe: Is it appropriate for people with progressive conditions? When do you considered the service should be provided? Who should provide it? Can you tell me the least criteria for the composure of the support team?
3. Can you tell me how home based palliative care is appropriate for people with cancer? Probe: how do you enroll patients to the program? What are the barriers to enroll patients for home based palliative care service?
4. Are you able to respond to patient’s palliative care? Probe: How is your relationship with the patients? How close are you? What, if any, challenges do you meet when taking care of adult patients with cancer?
5. Do you acknowledged and discussed with the patient and family on preferred place of care? Probing: Do you comply with thier preferences? What influences the place of care preference of patients? Are you involved in decision making process? How? When? What, if any, challenges do you meet?
6. What are the barriers for patients to get palliative care service? Probe: from health facilities; from health care providers; from your perspective?
7. What are the barriers for patients to get home based palliative care service? Probe: from health facilities, from health care providers, from your perspective;
8. Is there a link from health facility to home based care service for this patient’s? Probe: where should the link start, do you think there is a gap? Who should work on liaising patients?
9. Do you discuss the need for availability of continuity of palliative care with patients and families? What, if any, challenges do you meet? What are the barriers?
10. What do you need to be able to increase your competencies in PC? Probe: what adequate prerequisites do you have for effective communication (i.e. training and education, as well as access to updated information and technology?) What, if any, challenges do you meet?
11. What are the barriers for continuity of palliative care from facility to household and from household to facility after informing prognosis of the disease? Probe: How do you go about to the next step? Do you initiate discussion about the future?
12. Anything we didn’t mention but missing and want to discuss take the time?

**Thank you for your kind cooperation, I will re-visit you based on your willingness for missed or untouched issues, if any.**
