# Supplementary material for: Barrier analysis for continuity of palliative care from health facility to household among adult cancer patients in Addis Ababa, Ethiopia
Source: BMC Palliat Care. 2023 May 12;22:57. doi: 10.1186/s12904-023-01181-w (PMC10175902; doi:10.1186/s12904-023-01181-w)
Supplement: Supplementary file 4 — Additional file 4: Interview guide for policymakers. The interview guide includes questions on socio-demographic characteristics, and questions about diagnosis, barriers to palliative care, continuum of care, and areas of improvement. [file 12904_2023_1181_MOESM4_ESM.docx]

Interview guide for Policymakers

1. Can you please Introduce yourself? Probe; Age, Educational, Marital status, Employment status, Experience in the area?
2. What is your contribution/involvement in palliative care? Probe: For palliative care providing facilities, policy development?
3. Is there a National/Region specific strategy for the implementation of palliative care provision? Probe: Is the policy operational, underdevelopment, or not in effect? What is planned?
4. Can you explain how the continuum of palliative care is active in the current health system? Who are involved and at what stage? What has been done so far?
5. Can you tell me about the drug or medication in primary health care units? Probe: Are they available year-long? What about Morphine production and transaction in our country? What has been done about the spiritual, Psycho-social components of palliative care?
6. How do you safeguard the continuity of palliative care from health facility to household? Probe: Infrastructure for providing home-based palliative care services in the community? Palliative care education? Implementation? What are the challenges and the solution?
7. What are the barriers at your level for the nexus of palliative care from Health-facility to household level? Probe: what barriers have you ever faced before what solution did you offer; and was the solution effective?
8. Do you have monitoring and evaluation? Who are to be monitored and evaluated? Based on what? Do you initiate discussion about the future? Does the legislation have a regular review to improve access to palliative care?
9. So finally what should be done to improve palliative care service and the continuum of palliative care?
10. Anything we didn’t mention but are missing and want to discuss take the time?

**Thank you for your kind cooperation, I will re-visit you based on your willingness for missed or untouched issues if any.**
